# Pulsed Electromagnetic Fields Enhance Resveratrol-Induced Apoptosis Through Modulation of Apoptotic Signaling in U87-MG Glioblastoma Cells

**DOI:** 10.3390/ijms27187986

**Published:** 2026-09-08

**Authors:** Sinan Kandir, Atike Dogan, Kayhan Ates, Sukru Ozen, Serdar Karakurt, Cigdem Gokcek-Sarac

**Affiliations:** 1 Department of Veterinary Physiology, Faculty of Ceyhan Veterinary Medicine, Cukurova University, Adana 01250, Türkiye; sinankandir@cu.edu.tr; 2 Department of Biomedical Engineering, Faculty of Engineering, Akdeniz University, Antalya 07058, Türkiye; 3 Department of Electrical and Electronics Engineering, Faculty of Engineering, Akdeniz University, Antalya 07058, Türkiye; kayhanates@akdeniz.edu.tr (K.A.); sukruozen@akdeniz.edu.tr (S.O.); 4 Department of Biochemistry, Faculty of Science, Selcuk University, Konya 42130, Türkiye; karakurt@selcuk.edu.tr; 5 BioMediTech Institute, Faculty of Medicine and Health Technology, University of Tampere, FI-33014 Tampere, Finland

**Keywords:** glioblastoma, electromagnetic fields, resveratrol, apoptosis, PI3K/AKT/mTOR, PEMF

## Abstract

Glioblastoma multiforme (GBM) is an aggressive primary brain tumor characterized by dysregulated cellular signaling and resistance to apoptosis. Pulsed electromagnetic field (PEMF) exposure has been reported to modulate cancer-related cellular processes, while resveratrol (RES), a natural polyphenol, exhibits notable antiproliferative and pro-apoptotic actions. Here, we investigated whether 75 Hz PEMF potentiates RES-mediated apoptosis in human glioblastoma U87-MG cells by examining key molecules involved in apoptotic signaling and the transcriptional responses of PI3K/AKT/mTOR pathway-related genes. U87-MG glioblastoma cells and non-tumour human astroglial SVG-P12 cells were cultured and divided into the following six groups: (I) untreated cells; (II) RES-treated cells; (III) temozolomide (TMZ)-treated cells; (IV) PEMF-exposed cells; (V) cells treated with RES followed by PEMF exposure; and (VI) cells treated with TMZ followed by PEMF exposure. Cell viability was assessed in both cell lines by alamar blue assay, and selectivity indices were derived from the resulting IC50 values. Apoptotic responses and gene expression profiles were assessed in U87-MG cells by flow cytometry and qRT-PCR, while selected apoptosis-related proteins were evaluated by Western blotting. RES treatment induced stronger cytotoxic effects than TMZ in U87-MG cells, and this response was markedly enhanced following PEMF exposure, the IC_50_ falling from 33.64 to 10.53 µg/mL. In SVG-P12 astroglia the IC_50_ of RES rose from 17.95 to 98.31 µg/mL under the same exposure, so that the selectivity index of RES increased from 0.53 to 9.34, whereas that of TMZ remained essentially unchanged (1.54 versus 1.50). Combined RES and PEMF treatment significantly increased both early and late apoptosis. Molecular analyses revealed upregulation of pro-apoptotic markers accompanied by an increased Bax/BcL-2 ratio, consistent with protein-level changes. Transcripts of PI3K/AKT/mTOR pathway-related components were also elevated relative to untreated cells, but to a markedly lesser extent than the apoptotic markers. PEMF exposure significantly enhances the pro-apoptotic effects of RES in glioblastoma cells while relatively preserving SVG-P12 cell viability, supporting further investigation of this combination as a potential adjuvant approach.

## 1. Introduction

Cancer persists as a critical global health issue, with a predicted 20 million new cases and about 9.7 million deaths worldwide in 2022, according to GLOBOCAN estimates [1]. This underscores the urgent need to develop more effective diagnostic and therapeutic strategies. Glioblastoma multiforme (GBM) is a frequently diagnosed and extremely aggressive primary brain tumor in adults, representing 45.2% of malignant primary brain and central nervous system tumors and around 54% of all gliomas [2]. Despite modern treatments, it is still a deadly condition with invasive growth, rapid proliferation, and a high chance of recurrence [3,4]. Temozolomide (TMZ) is an alkylating chemotherapeutic agent administered in combination with radiotherapy following surgical resection for the treatment of GBM. However, resistance to TMZ is still one of the critical problems limiting treatment efficacy [5].

Electromagnetic fields (EMFs) result from the interaction of electric and magnetic fields and are generally classified as either “higher-frequency EMFs” or “low-to-mid-frequency EMFs”. High-frequency EMFs, which contain the non-ionizing radiation part of the electromagnetic spectrum, can cause adverse biological effects such as induction of DNA damage and oxidative stress [6]. In contrast, low- to mid-frequency EMFs have a non-ionizing interaction mechanism with biological systems and are generally associated with milder biological effects, some of which may even have therapeutic potential [6,7,8]. Pulsed Electromagnetic Fields (PEMFs) are considered one of the most remarkable low-frequency EMFs that serve as a non-invasive and safe therapeutic approach [9,10]. Unlike high-frequency EMFs, PEMFs belong to the non-ionizing spectrum and interact with biological systems without inducing thermal effects [6,11,12]. PEMFs exert their effects at the cellular level through different mechanisms including the modulation of intracellular signaling pathways [13], regulation of oxidative stress [12,14], and influence on the apoptotic mechanisms in cancer cells [15,16].

In GBM, apoptosis is primarily mediated through the intrinsic (mitochondrial) and extrinsic (death receptor) pathways, whereas the phosphatidylinositol 3-kinase (PI3K)/AKT/mammalian target of rapamycin (mTOR) pathway mainly functions as a pro-survival signaling cascade that suppresses apoptotic cell death. Within the intrinsic apoptotic pathway, pro-apoptotic proteins (Bax, BcL-xS, Bad, Bim, Bid) and members of the BcL-2 family with anti-apoptotic functions, such as BcL-2, BcL-xL, and Mcl-1, are primary factors determining cell survival or death. Pro-apoptotic proteins such as Bax and Bid promote the release of cytochrome c from mitochondria into the cytosol, initiating apoptosis, whereas anti-apoptotic proteins prevent this release and thereby inhibit apoptosis [17,18]. Within the extrinsic death receptor pathway, TNF-α and its receptor TNFR1 initiate downstream signaling events that regulate apoptosis-related gene expression. This event subsequently triggers the movement of NFκB into the cell nucleus, where the transcription of several genes linked to apoptosis is altered, including caspase-7, caspase-9, and caspase-3. The PI3K/AKT/mTOR pathway is an important intracellular route that controls the cell cycle. It can be turned on by a number of stimuli, such as insulin, glucose, cytokines, growth factors, and other chemical signals [19]. In GBM, this pathway inhibits apoptosis [20]. PI3K is a central kinase that activates critical signaling pathway components in the PI3K-AKT-mTOR pathway that control cell development, motility, and apoptosis [21,22,23]. One of these components is mTOR, the mammalian target of the rapamycin protein complex [24]. mTOR inhibits the activation of signal transduction target molecules such as p53, BcL-2, BcL-2-antagonist (BAD), p21, p27, c-myc, which regulate apoptotic cell death [25,26]. AKT, a serine/threonine kinase, regulates PI3K signaling, which in turn controls mTOR activity and inactivates pro-apoptotic factors like caspase-9 and Bad when it is phosphorylated at Thr308 and Ser473 [27]. The dysregulation of the PI3K/AKT/mTOR signaling pathway has a role in the progression of multiple cancer types [28]. Approximately 80% of GBM cases exhibit aberrant PI3K/AKT signaling, with phosphorylated AKT (pAKT) detected in 84% and a strong correlation with increasing AKT activation and pathological severity [20,29,30]. Inhibition of PI3K/AKT/mTOR pathway enhances apoptosis and presents a promising therapeutic strategy for GBM [26].

Resveratrol (RES), a natural polyphenol with a stilbene backbone, was initially isolated in 1940 from *Veratrum grandiflorum O. Loes* (white hellebore) roots and it has garnered growing attention in the fields of medicine and pharmacology [31,32]. RES possesses several therapeutic effects, including anti-cancer [33,34], anti-aging [35], anti-inflammatory [36,37], anti-viral [38], and cholesterol-lowering actions [39]. It influences critical processes including cell proliferation, inflammation, apoptosis, metastasis, and angiogenesis by targeting several signaling pathways, consequently impacting numerous phases of cancer formation, such as initiation, promotion, and progression [33,40].

Despite its promising anticancer properties, the therapeutic translation of RES is challenged by its limited bioavailability and by the relatively high concentrations frequently required to reproduce its biological effects in experimental settings [31,34,40]. In contrast, PEMF represents a non-invasive physical approach capable of modulating intracellular signaling, oxidative stress, and apoptosis without introducing an additional pharmacological agent [13,15]. These complementary characteristics provide a biological rationale for combining PEMF exposure with RES treatment. In particular, PEMF-induced modulation of cellular processes may increase the responsiveness of glioblastoma cells to RES, thereby potentially enhancing its cytotoxic and pro-apoptotic effects at lower RES concentrations. Thus, the combination of RES with PEMF may represent a promising strategy to enhance the antitumor effects of RES while potentially improving tumor-cell selectivity. Research investigating the combined effects of PEMF and RES in brain cancer remains limited, particularly with respect to their effects on apoptosis-related signaling mechanisms in GBM. Specifically, there is a notable gap in studies examining the effects of PEMF exposure together with RES on pro-apoptotic signaling and PI3K/AKT/mTOR gene expression in human glioblastoma cells. Therefore, we aimed to elucidate the effects of combined PEMF exposure and RES treatment on apoptotic mechanisms in human U87-MG glioblastoma cells by assessing pro-apoptotic markers at the gene and protein levels and the transcriptional responses of PI3K, AKT, and mTOR.

## 2. Results

**Figure 1:**
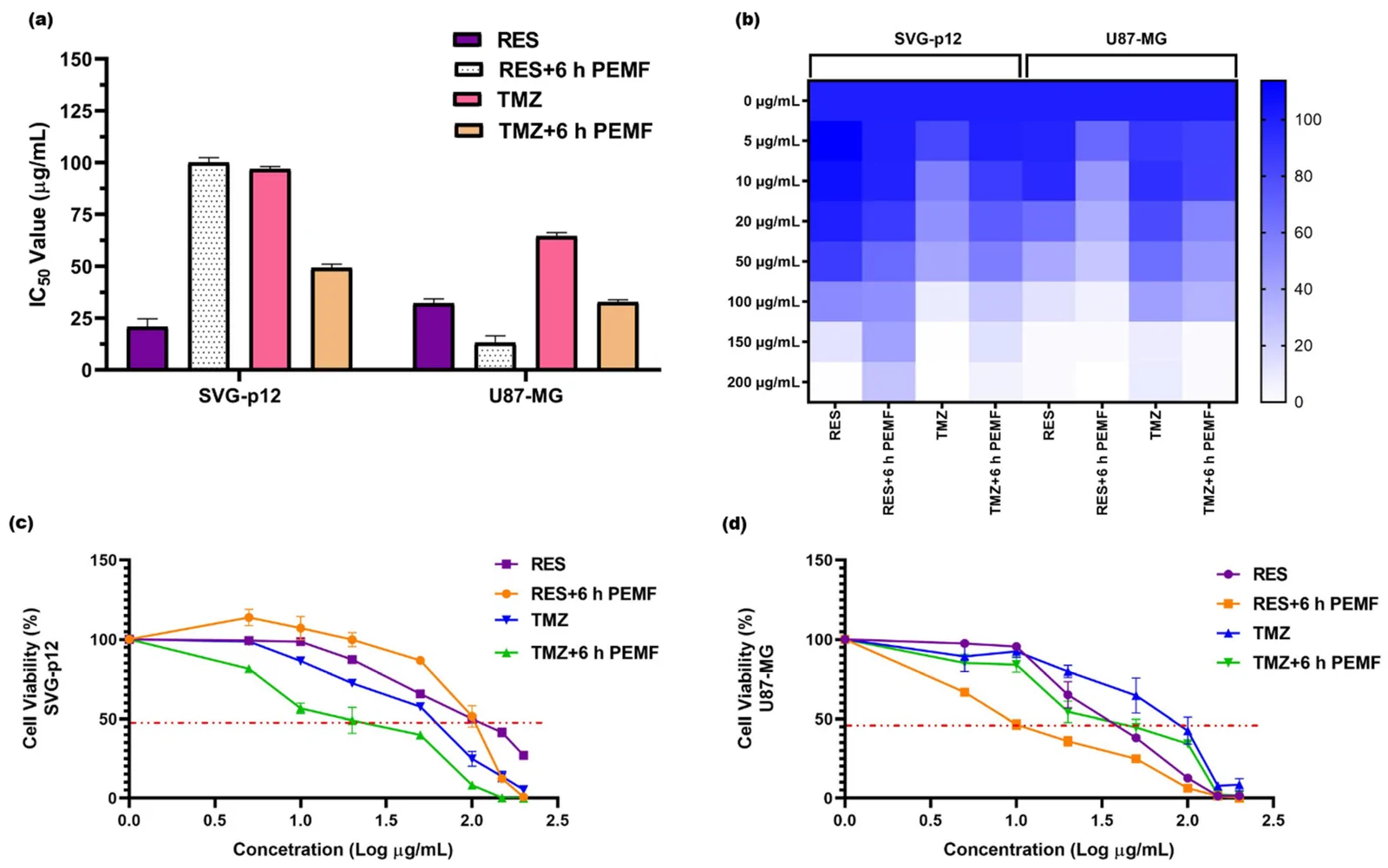
Cytotoxic effects of RES, TMZ, and their combination with 6 h PEMF exposure on healthy astroglial SVG-P12 and glioblastoma U87-MG cell lines. (**a**) IC_50_ values of RES and TMZ, with or without 6 h PEMF exposure at 75 Hz, were determined in U87-MG and SVG-P12 cell lines by the Alamar Blue assay. (**b**) Heatmap representation of dose-dependent cell viability in SVG-p12 and U87-MG cells treated with elevated concentrations (0–200 µg/mL) of RES and TMZ, with or without PEMF. (**c**) Percentage of cell viability in SVG-p12, and (**d**) in U87-MG cells treated with RES, TMZ, or their PEMF combinations. The result is shown as mean ± SD from three independent trials carried out in triplicate.

Cytotoxicity analyses were carried out to assess the responses of cells to RES and TMZ treatment, with TMZ applied as the reference agent. Cells were treated with increasing concentrations of RES and TMZ (0–200 µg/mL), subjected to 6 h PEMF or sham exposure as appropriate, and then incubated for an additional 48 h before cell viability assessment. IC_50_ values were subsequently calculated in human glioblastoma U87-MG and healthy human astroglia SVG-P12 cell lines, under conditions with or without 6 h PEMF exposure. In U87-MG cells without PEMF exposure, IC_50_ values for RES and TMZ were 33.64 µg/mL and 63.27 µg/mL, respectively. In contrast, these values were reduced to 10.53 µg/mL and 31.83 µg/mL in cells exposed to PEMF for 6 h (Figure 1a). In healthy human astroglia SVG-P12 cells, the IC_50_ values for RES and TMZ without PEMF exposure were 17.95 µg/mL and 97.70 µg/mL, respectively. PEMF exposure shifted these values to 98.31 µg/mL and 47.90 µg/mL (Figure 1a).

Consistent with IC_50_ results, cell viability assays confirmed that PEMF exposure increased SVG-P12 cell viability when combined with resveratrol, whereas in U87-MG glioblastoma cells, PEMF significantly enhanced the cytotoxic impact of both RES and TMZ (Figure 1c,d). Expressed as a selectivity index (SI), defined as the IC_50_ in SVG-P12 divided by the IC_50_ in U87-MG, RES alone showed no preference for tumour cells (SI = 0.53), whereas RES combined with 6 h PEMF exposure reached an SI of 9.34. The corresponding values for TMZ were 1.54 without and 1.50 with PEMF exposure, indicating that this shift in selectivity was specific to the RES and PEMF combination.

By examining IC_50_ values to determine cell viability, RES demonstrated a more potent cytotoxic effect than TMZ in U87-MG cell lines, both in PEMF-exposed and non-exposed conditions. Furthermore, the cytotoxic effects of both RES and TMZ were significantly greater in the PEMF-exposed U87-MG cells than in the non-exposed cells (Figure 1d). In SVG-P12 cells, RES exhibited greater cytotoxicity than TMZ in the absence of PEMF exposure. Following PEMF exposure, RES-induced cytotoxicity decreased, whereas TMZ-induced cytotoxicity increased (Figure 1c).

Heatmap analysis provided a qualitative visualization of the dose-dependent changes in cell viability across the treatment groups (Figure 1b). Quantitative comparisons of cytotoxic potency were based on the calculated IC_50_ values and the corresponding dose–response analyses rather than on visual estimation of concentration ranges from the heatmap color scale.

After carefully examining these findings, PEMF exposure may modulate the cytotoxic effects of RES and TMZ in a cell type-specific manner. In SVG-P12 cells, PEMF exposure reduced RES-induced cytotoxicity but increased TMZ-induced cytotoxicity. In contrast, PEMF exposure increased the cytotoxic effects of both RES and TMZ in U87-MG cells. Notably, the marked increase in the selectivity index observed for RES following PEMF exposure suggests a favorable selectivity profile for the RES-PEMF combination under the present experimental conditions.

**Figure 2:**
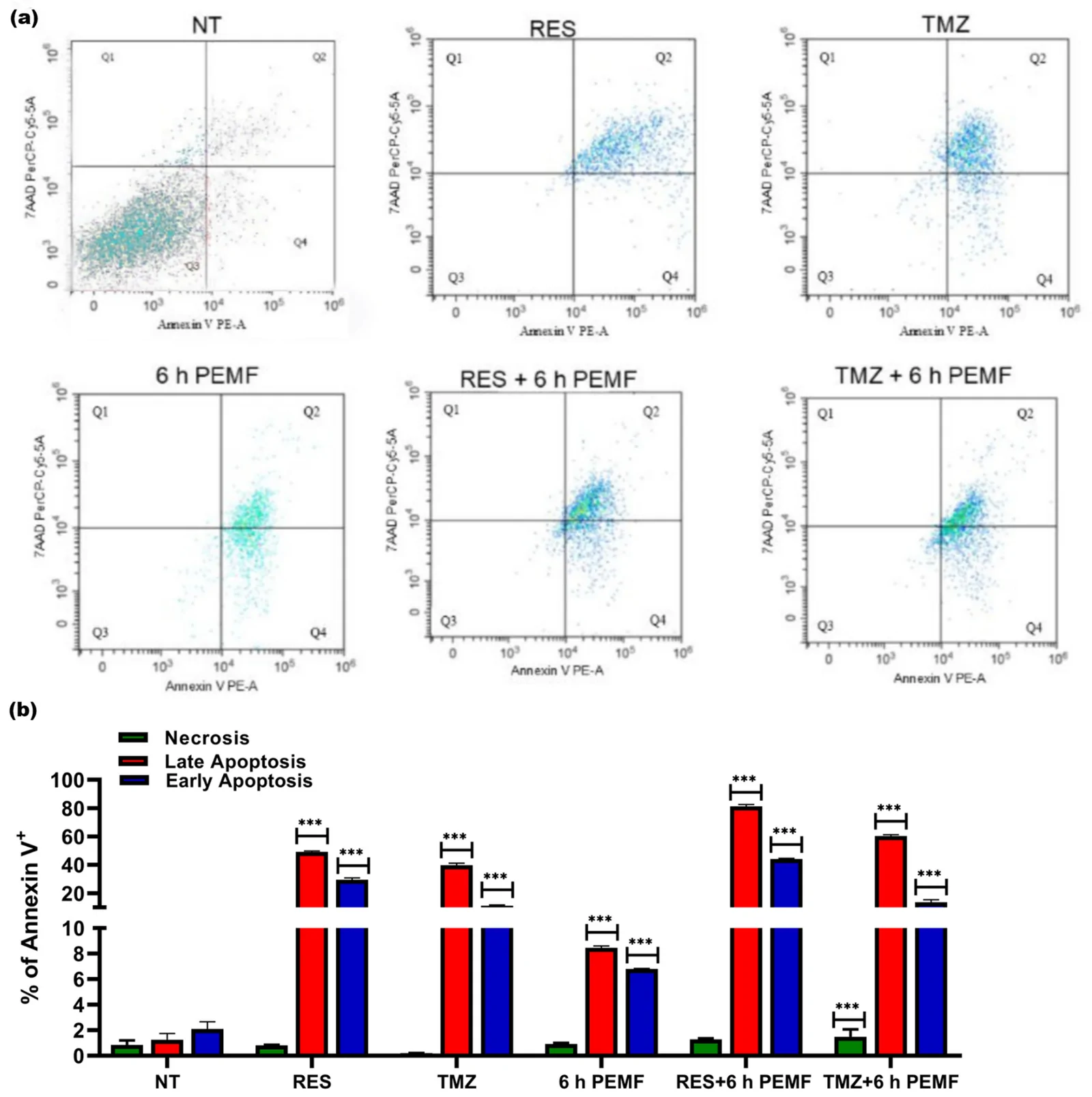
Flow cytometric analysis of apoptosis in U87-MG cells treated with RES and TMZ, alone or followed by 6 h exposure to a 75 Hz PEMF. (**a**) Representative Annexin V/7-AAD flow-cytometry dot plots of all experimental groups: NT, RES, TMZ, 6 h PEMF, RES followed by 6 h PEMF, and TMZ followed by 6 h PEMF. Quadrants represent necrotic cells (Q1: Annexin V−/7-AAD+), late apoptotic cells (Q2: Annexin V+/7-AAD+), viable cells (Q3: Annexin V−/7-AAD−), and early apoptotic cells (Q4: Annexin V+/7-AAD−). (**b**) Quantitative analysis of necrotic, early apoptotic, and late apoptotic cell populations. Data are presented as mean ± SD from three independent experiments, each performed with three technical replicates. Statistical significance versus the NT group is indicated as *** *p* < 0.001.

Flow cytometry analyses were performed to evaluate apoptosis and necrosis to clarify whether the observed cytotoxicity was associated with apoptotic mechanisms (Figure 2a). The apoptotic response was more pronounced in the groups treated with RES or TMZ followed by 6 h PEMF exposure than in the corresponding treatment-alone groups. Specifically, late apoptosis was 81.35% in the group treated with RES followed by 6 h PEMF exposure, and 60.43% in the group treated with TMZ followed by 6 h PEMF exposure, compared with 49.09% in the RES-only group, and 39.68% in the TMZ-only group. The late apoptosis value was 8.46% in the PEMF-only group (Figure 2b). Similarly, early apoptosis was 44.08% in the group treated with RES followed by 6 h PEMF exposure and 13.71% in the group treated with TMZ followed by 6 h PEMF exposure, compared with 29.52% in the RES-only group and 11.24% in the TMZ-only group. The early apoptosis value was 6.81% in the PEMF-only group (Figure 2b). Overall, the most pronounced apoptotic response was observed in the group treated with RES followed by PEMF exposure.

**Figure 3:**
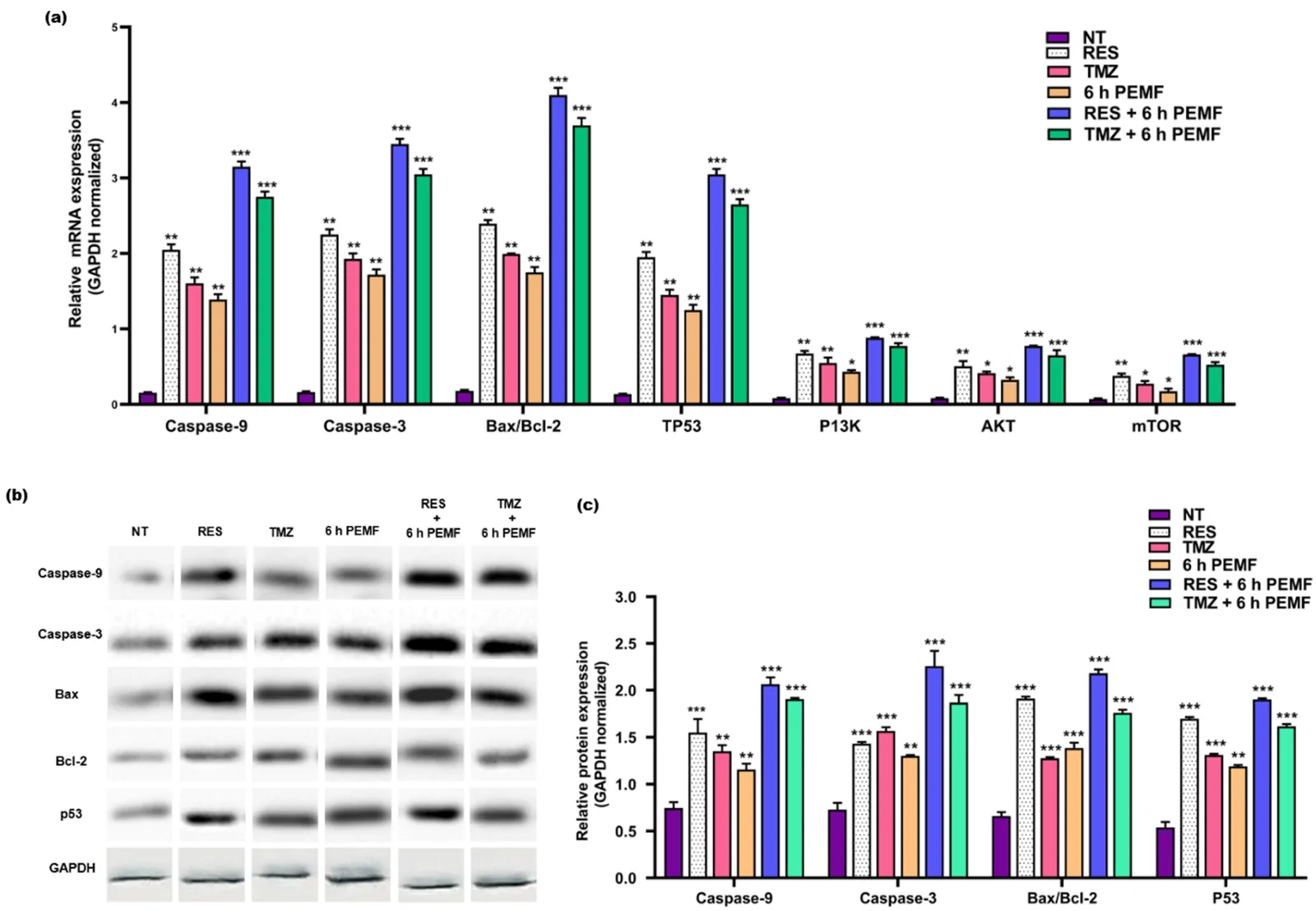
Gene expression and apoptosis-related protein expression profiles in human glioblastoma U87-MG cells. (**a**) Quantitative real-time PCR analysis of key genes in U87-MG cells treated with RES or TMZ, alone or combined with 6 h PEMF exposure. Expression levels were normalized against GAPDH as an internal control. (**b**) Representative immunoblots acquired from the same membrane for each target protein, with GAPDH used as a loading control. All bands were cropped from the same exposure; non-adjacent lanes are separated by thin dividers. (**c**) Densitometric quantification of protein bands normalized to GAPDH. The data are expressed as mean ± SD from three independent trials, each performed in triplicate. Statistical significance in comparison to the non-treated (NT) group is indicated as follows: * *p* < 0.05, ** *p* < 0.01, *** *p* < 0.001.

To elucidate the molecular mechanisms behind apoptosis induced by PEMF and RES treatment, the expression profiles of critical pro-apoptotic genes and genes linked to the PI3K/AKT/mTOR pathway were analyzed using qRT-PCR. As shown in Figure 3a, mRNA levels of caspase-9, an initiator caspase involved in the intrinsic apoptotic pathway, and caspase-3, a key executioner caspase involved in both intrinsic and extrinsic apoptotic processes, were significantly upregulated in the group treated with RES followed by 6 h PEMF exposure relative to the NT group (*** *p* < 0.001, respectively). In this group, caspase-9 and caspase-3 gene expressions increased by approximately 1.5-fold compared with RES treatment alone. Similarly, in the group treated with TMZ followed by 6 h PEMF exposure, caspase-9 and caspase-3 levels were also markedly elevated relative to the NT group (*** *p* < 0.001, respectively), with increases of approximately 1.7-fold and 1.5-fold, respectively, compared with TMZ treatment alone. Notably, these genes showed about 1.5-fold lower expression in the TMZ-treated group following 6 h PEMF exposure than in the group receiving RES followed by the same PEMF exposure.

The ratio of Bax, a pro-apoptotic gene, to BcL-2, an anti-apoptotic gene, was highest in the group treated with RES, followed by 6 h PEMF exposure, in comparison with NT group (*** *p* < 0.001), exhibiting a 1.71-fold increase relative to RES treatment alone. A similar, though less pronounced, elevation was observed in the group treated with TMZ followed by 6 h PEMF exposure (*** *p* < 0.001), where the Bax/BcL-2 ratio was approximately 1.3-fold lower than in the group treated with RES followed by 6 h PEMF exposure, but nearly 2-fold higher than in the group treated with TMZ alone.

The significantly increased TP53 mRNA expression observed in the RES treated group, followed by 6 h PEMF exposure, compared to the NT group (*** *p* < 0.001), suggests the activation of apoptotic processes. This increase was 1.56 times higher than in the group treated with RES alone. The group treated with TMZ followed by 6 h of PEMF exposure exhibited a significant increase in TP53 expression compared to the NT group (*** *p* < 0.001), with expression levels approximately twice as high as those observed in the group treated with TMZ alone. However, TP53 gene expression was approximately 1.2-fold lower in the group treated with TMZ, followed by 6 h PEMF exposure, compared to the group treated with RES, followed by 6 h PEMF exposure.

When PI3K/AKT/mTOR pathway-related genes were analyzed, transcript levels of PI3K, AKT and mTOR were significantly elevated relative to the NT group in cells treated with RES followed by 6 h PEMF exposure (*** *p* < 0.001, respectively). However, the overall magnitude of the transcript-level increases in PI3K, AKT, and mTOR was approximately 4-fold lower than that observed for the key genes involved in apoptosis. Similarly, in the group treated with TMZ followed by 6 h PEMF exposure, PI3K, AKT and mTOR transcript levels were also significantly higher in comparison with the NT group (*** *p* < 0.001, respectively), but this increase was lower than that observed in the RES-treated group followed by 6 h PEMF exposure, and approximately 5.2-fold lower in overall magnitude than that observed for the apoptosis-related genes. These findings describe the relative magnitude of the transcriptional responses and should not be interpreted as evidence of functional activation or suppression of the PI3K/AKT/mTOR pathway.

To determine whether the transcriptional changes observed in the pro-apoptotic markers were also reflected at the protein level, Western blotting was performed to evaluate selected apoptosis-related proteins (Figure 3b). According to the results, the expression of all studied proteins was markedly higher in the group treated with RES, followed by 6 h PEMF exposure in comparison to the NT group (*** *p* < 0.001, respectively). In this group, caspase-9, caspase-3, Bax/BcL-2, and P53 protein expressions increased approximately 1.4-fold, 1.6-fold, 1.2-fold, and 1.1-fold, respectively, relative to RES treatment alone. Similarly, the expressions of the same proteins in the group treated with TMZ followed by 6 h PEMF exposure were significantly elevated in comparison with the NT group (*** *p* < 0.001, respectively), corresponding to approximately 1.5-fold, 1.2-fold, 1.5-fold, 1.2-fold increases, respectively, compared with TMZ treatment alone (Figure 3c). Notably, the expression levels of all proteins were approximately 1.2-fold lower in the group treated with TMZ, followed by 6 h PEMF exposure than in the group treated with RES, followed by 6 h PEMF exposure.

## 3. Discussion

In recent years, low-frequency EMFs that do not exhibit ionizing or thermal effects have been increasingly investigated for their potential therapeutic effects in medicine, with the most remarkable being the application of PEMF, characterized by a specific waveform and amplitude [41]. Low-frequency PEMF exposure has a wide range of applications, such as facilitating wound repair [42], improving soft tissue damage [43], reducing edema [44], treating migraines [45], and having potential in the treatment of neurodegenerative disorders like Alzheimer’s [46], and Parkinson’s [47], while also providing neutralization in tissues by periodically stimulating cells with rhythmic waves, modulating the oxidant-antioxidant balance, enhancing cell membrane permeability, reducing oxidative stress, and inducing apoptosis in cancer cells [15,48,49,50]. Non-invasive electromagnetic-based strategies have received attention as potential adjuvant to cancer therapy because of their capacity to enhance tumor cell sensitivity while reducing systemic toxicity.

The literature shows a notable lack of studies examining the combined effects of PEMF exposure and RES on the apoptotic pathway in GBM. Hence, in this study, we aimed to expose RES-treated human U87-MG glioblastoma cells to 75 Hz PEMF to investigate the potential effects of the RES-PEMF combination on cell viability, as well as on the expression of key pro-apoptotic markers and PI3K/AKT/mTOR-related transcripts. To enable comparison with current treatment modalities, TMZ was included in the study groups as a positive control.

When the cytotoxicity data obtained from the study were collectively evaluated, it was observed that RES exhibited a more potent cytotoxic effect than TMZ, with a significant decrease in IC_50_ values in the human glioblastoma U87-MG cell line. This effect was further enhanced by PEMF exposure. In contrast, in the healthy astroglial SVG-P12 cell line, RES exhibited greater cytotoxicity than TMZ in the absence of PEMF exposure. Following PEMF exposure, RES-induced cytotoxicity decreased markedly, whereas TMZ-induced cytotoxicity increased. Thus, PEMF exposure produced opposite effects on RES and TMZ responses in SVG-P12 cells. TMZ exerts its antitumor effects by crossing the blood–brain barrier and inducing DNA damage in rapidly proliferating glioblastoma cells. The combination of RES, known for its anti-cancer, anti-inflammatory and antioxidant properties, with PEMF showed a more pronounced cytotoxic effect in U87-MG cells, and this enhancement may be related to RES’s capacity to modulate multiple signaling pathways involved in cell viability, proliferation, and apoptosis. These findings suggest that PEMF exposure modulates the response to both RES and TMZ in a cell type-specific manner, with enhanced cytotoxicity in U87-MG cells and relative preservation of SVG-P12 cell viability. This selective enhancement of cytotoxicity in glioblastoma cells, along with the relative preservation of viability in healthy astroglial cells, may represent a favorable selectivity profile compared with TMZ under the present experimental conditions. Expressed as selectivity indices, the values recorded here also compare favorably with published data for RES in other tumor models. In head and neck carcinoma lines, RES alone yielded selectivity indices of 2.15 and 2.41 against normal keratinocytes, values regarded by those authors as indicative of high selectivity [51]. In the present study RES alone showed no preference for glioblastoma cells (SI = 0.53), whereas the addition of 75 Hz PEMF raised the index to 9.34, indicating that the physical stimulus rather than the polyphenol itself accounted for the shift in selectivity.

To elucidate the mechanisms behind this cytotoxicity, we further investigated the apoptotic responses triggered by RES and TMZ treatments, both alone and in combination with 6 h 75 Hz PEMF exposure by flow cytometry. In comparison to RES or TMZ treatment alone, it was observed that 6 h PEMF exposure following RES or TMZ treatment markedly increased apoptosis in glioblastoma cells. Notably, this increase was more pronounced when PEMF was applied after RES treatment than TMZ treatment. These findings imply that the combination of PEMF and RES has a more pronounced pro-apoptotic effect than TMZ alone, although further validation in TMZ-resistant glioblastoma models is necessary to evaluate the wider therapeutic potential. Our findings are consistent with earlier research showing that exposure to PEMF with different frequencies and wavelengths, either alone or in combination with chemotherapeutic drugs, induces apoptosis in many types of glioblastoma cell lines by decreasing cell viability and inhibiting cell proliferation [52,53,54].

When we analyzed the gene and protein expression levels, particularly focusing on apoptotic markers, we observed significant changes, especially in the group treated with RES, followed by 6 h of 75 Hz PEMF exposure. The significantly higher expression of caspase-9 and caspase-3 in this group compared to the NT group supports the critical roles of these caspases in the apoptosis process. Caspase-9, an intrinsic caspase, is activated in response to cellular stress through the mitochondrial release of cytochrome c, which subsequently triggers the activation of caspase-3, the executioner caspase, ultimately leading to cell death in a regulated manner [55]. The combined effect of RES and PEMF may contribute to the increase in these caspases. These findings are consistent with enhanced apoptotic signaling following RES treatment and PEMF exposure, although the upstream mechanisms responsible for this response were not directly investigated. In parallel with the observed changes in gene and protein expression levels of caspase-9 and caspase-3, a marked increase in the Bax/BcL-2 ratio was observed in the group exposed to 75 Hz PEMF for 6 h after RES treatment. The pro-apoptotic protein Bax assists the caspase cascade activation by enhancing mitochondrial membrane permeability, which initiates the cytochrome c release. In contrast, the anti-apoptotic protein BcL-2 helps maintain mitochondrial membrane integrity. It inhibits apoptosis by preventing cytochrome c release [55]. The higher Bax/BcL-2 ratio observed in the PEMF-exposed group following RES treatment may indicate a shift toward pro-apoptotic signaling that favors the initiation of cell death, potentially triggered by DNA damage, cellular stress, or toxic effects, suggesting that PEMF exposure may sensitize glioblastoma cells to apoptosis.

If DNA damage occurs, the p53 gene arrests the cell cycle at the end of the G1 phase, allowing for DNA repair. If DNA damage is effectively repaired, p53 levels fall, allowing the cell cycle to complete. Otherwise, if repair attempts fail, p53 initiates apoptosis in the cell [56,57]. The significant increase observed in TP53 mRNA and P53 protein levels in this combination group in our study is consistent with this mechanism. These findings suggest that this tumor suppressor gene is involved in the apoptotic response following RES treatment and subsequent PEMF exposure and are consistent with a more pronounced apoptotic response in glioblastoma cells. However, cellular stress was not directly assessed in the present study.

Our findings align with earlier studies reporting that applying PEMF with different frequencies and wavelengths at certain time intervals, either directly or in combination with chemotherapeutic drugs, to different glioblastoma cell lines alters cell viability, induces apoptosis by suppressing cancer cell proliferation, and causes changes in the levels of apoptosis and apoptosis-related genes. For example, exposing human U87, T98G, H4 GBM cell lines to different frequencies and intensities of ELF-PEMF (100 Hz, 10 mT; 10 Hz, 5 mT or 7 Hz, 30 mT) for different durations (12, 24, and 48 h), either directly or in combination with chemotherapeutic agents such as TMZ, carboplatin, has been shown to induce overexpression of caspase-3, Bax, and P53 proteins, reduce BcL-2 and cyclin-D1, promote cell differentiation, alter cell viability, and trigger apoptosis along with apoptosis-related morphological changes [52,53,54,58]. In addition to these aforementioned cellular effects, low-frequency pulsed magnetic fields (LF-PEMFs) have been shown to increase sensitivity to TMZ in U87, T98G, and C6 glioma cell lines through mechanisms such as induction of oxidative stress and promotion of cellular differentiation, contributing to the inhibition of tumor growth [59,60,61]. The tumor-suppressive effects of long-term LF-PEMF exposure have also been demonstrated, with a significant decrease in tumor volume in in vivo xenograft models [4]. Specific LF-PEMF parameters (60 Hz, 5 mT) have been reported to inhibit cell migration and cause DNA damage in A172 and U251 glioblastoma cells when applied with genotoxic agents such as methyl methane sulfonate (MMS) or hydrogen peroxide (H_2_O_2_) [4,62]. All these studies support the idea that PEMF applications at specific frequencies, doses, amplitudes, and durations have significant potential as an alternative therapeutic approach in the treatment of glioblastoma, highlighting the roles of PEMF applications in regulating basic cellular pathways that play a role in tumor progression and treatment resistance [4].

In the next phase of our study, we addressed how the combination of RES and PEMF exposure is associated with transcriptional changes in cellular survival pathways, in particular the PI3K/AKT/mTOR signaling cascade, which is a well-established regulator of cellular survival, proliferation, and motility in glioblastoma, playing a crucial role in apoptosis inhibition [20]. Specifically, transcript levels of PI3K, AKT, and mTOR were approximately 4-fold lower than those of the key pro-apoptotic genes, whereas protein expression analysis was limited to pro-apoptotic markers. Because the functional output of this cascade is governed principally by phosphorylation rather than by transcript abundance, the observed transcriptional changes in PI3K/AKT/mTOR pathway-related genes cannot be taken to reflect pathway activation or suppression directly. Thus, the present findings indicate that the magnitude of the transcriptional response of PI3K, AKT, and mTOR was comparatively limited relative to that of the pro-apoptotic markers, without establishing the functional status of the pathway. While the PI3K/AKT/mTOR pathway typically supports cellular survival and proliferation, the comparatively limited induction of its transcripts relative to the pro-apoptotic markers should therefore be interpreted only at the transcriptional level. Although literature suggests that PEMF can induce mTOR phosphorylation in specific cell types, such as osteoblasts and fibroblasts [63], and that intermittent PEMF exposure may sustain mTOR activation and promote cell proliferation [64], direct comparison with our findings is limited because phosphorylated and total AKT and mTOR were not evaluated in the present study. Accordingly, the observed changes in PI3K, AKT, and mTOR mRNA levels should be regarded as transcriptional responses accompanying RES and PEMF treatment rather than as evidence of a dominant activation or suppression of the PI3K/AKT/mTOR pathway.

### Limitations of the Study

The PI3K/AKT/mTOR cascade was assessed only at the transcript level. Because the functional output of this pathway is governed primarily by phosphorylation rather than by transcript abundance, the present data describe the relative magnitude of transcriptional responses and do not establish the activation status of the pathway; immunoblotting for phosphorylated and total AKT and mTOR, together with pharmacological inhibition of the cascade, would be required for that purpose. In addition, the molecular basis of the selectivity observed between glioblastoma and astroglial cells was not addressed, because gene and protein expression analyses were restricted to U87-MG cells. Although the current investigation is restricted to in vitro models, it provides preliminary evidence supporting further evaluation of PEMF as a non-invasive adjuvant approach in glioblastoma research. In order to further define the clinical translational potential of this strategy, future studies will be necessary to incorporate in vivo validation. In addition, additional examination of PEMF parameters—including dosage, frequency, intensity, amplitude, and exposure duration—in various cells, tissues, or animal models would enhance the acquisition of accurate scientific data regarding the physiological effects of PEMF. Therefore, the present study also guides further research focusing on the molecular, biochemical, and cellular processes activated by PEMF exposure.

## 4. Materials and Methods

### 4.1. Experimental Application of Pulsed Electromagnetic Fields

Pulsed electromagnetic field exposure was delivered using a programmable Pasco, UI-5000 setup (Pasco Scientific, Roseville, CA, USA), purchased from Edutek Company in Ankara, Turkiye. The device is capable of generating different waveforms, with a power output of up to 15 W and operates across a frequency range from DC to 100 kHz, with a resolution of 1 mHz. For the present experiments, a square-wave signal was generated using Pasco Capstone software (version 2.0, Pasco Scientific, Roseville, CA, USA), with the pulse frequency set to 75 Hz, a pulse duration of 1.3 milliseconds corresponding to a duty cycle of 1/10, and a signal amplitude of 15 V. A pair of 500-turn Helmholtz coils was arranged in parallel to create a uniform magnetic field for cell culture exposure. The Helmholtz coils were positioned with a 5 cm gap, and the cell cultures were placed within the uniform field region between the coils. A Hall effect-based sensor (CI-6520A, Pasco Scientific, Roseville, CA, USA) with 0.2% accuracy was used to measure peak magnetic field values, ensuring a stable magnetic flux density of 1 ± 0.2 mT between the two coils in the system where the cultures were placed. The entire exposure system was enclosed in a custom-built glass cabinet designed to maintain controlled cell-culture conditions during PEMF exposure. The temperature was maintained at 37 °C using a heating system with continuous temperature monitoring, while the CO_2_ concentration and relative humidity were maintained at 5% and approximately 95%, respectively. The exposure system represents a modified version of a previously utilized setup [15], with improved environmental control and signal stability. Further details of the exposure-system geometry, magnetic field measurements, and numerical dosimetry simulations are provided in the Supplementary Material [65,66], where Figure S1a presents the overall exposure system layout, Figure S1b shows its schematic with dimensions, and Figure S2 presents the numerical dosimetry simulation of the magnetic flux density distribution.

### 4.2. In Vitro Evaluation of Cytotoxic Effects

#### 4.2.1. Cell Culture Procedures

Human U87-MG glioblastoma and healthy human brain astroglia cell line SVG-p12 (ATCC, Manassas, VA, USA) were cultured in Dulbecco’s Modified Eagle’s Medium (DMEM, Thermo Fisher Scientific, Waltham, MA, USA) using the 75 cm [2] flasks (Corning, Corning, NY, USA). The culture medium consisted of inactivated fetal bovine serum (10% (*v*/*v*), FBS, Biological Industries, Cromwell, CT, USA), penicillin-streptomycin (1% (*v*/*v*), Pen-Strep, Biological Industries, Cromwell, CT, USA), and L-glutamine (2 mM, Biological Industries, Cromwell, CT, USA). Cells were incubated at 37 °C in a standard humidified environment containing 5% CO_2_. Upon transferring to 96-well plates, experimental groups of cells were settled as follows: Group 1: U87-MG cells without any treatment (non-treated-NT); Group 2: U87-MG cells treated with RES; Group 3: U87-MG cells treated with TMZ; Group 4: U87-MG cells exposed to PEMF at 75 Hz for 6 h; Group 5: U87-MG cells treated with RES, followed by 6 h exposure to 75 Hz PEMF; and Group 6: U87-MG cells treated with TMZ, followed by 6 h exposure to 75 Hz PEMF. The non-PEMF groups (Groups 1–3: NT, RES-treated, and TMZ-treated cells) underwent sham exposure using the same Helmholtz coil system within the same custom-built glass cabinet, but in a separate experimental run with no current supplied to the coils, and were maintained for 6 h under the same environmental conditions used for PEMF exposure. No second Helmholtz coil system was used. Group 1 served as the untreated sham control, whereas Groups 2 and 3 received RES and TMZ treatment, respectively. The PEMF-exposed groups (Groups 4–6) were positioned within the magnetic field system and each group was exposed separately in a distinct experimental run. Sham and PEMF-exposed groups were therefore not positioned simultaneously within the exposure system. The same six treatment groups were also applied to SVG-P12 astroglial cells for the assessment of cytotoxic responses, whereas apoptosis, gene and protein expression analyses were performed in U87-MG cells. U87-MG and SVG-P12 cells were exposed in separate experimental runs.

#### 4.2.2. Measurement of Cytotoxic Responses

Cells were trypsinized and stained with Trypan Blue (0.04%, 1:1 dilution, Sigma-Aldrich, St. Louis, MO, USA). Cell counts were obtained using an automatic cell counter (TC20, Bio-Rad, Hercules, CA, USA). Cells were distributed in triplicate onto 96-well culture plates (1.2 × 10^4^ cells/well) and incubated for 24 h at 37 °C. Once sufficient density was reached, cells were treated with RES and TMZ at 0, 5, 10, 20, 50, 100, 150, and 200 µg/mL. Following RES or TMZ treatment, the corresponding PEMF groups were exposed to 75 Hz PEMF for 6 h, whereas the non-PEMF groups underwent sham exposure for the same duration using the Helmholtz coil system with no current supplied to the coils, as described above. After completion of the 6 h PEMF or sham exposure, all groups were incubated for an additional 48 h in a sterile incubator (Binder, Camarillo, CA, USA) at 37 °C, 95% humidity, and 5% CO_2_. Cell viability was then assessed using the Alamar Blue Assay (Thermo Fisher Scientific, Waltham, MA, USA). After addition of the Alamar Blue reagent, cells were incubated for 3 h at 37 °C. Colorimetric changes were measured with a scanning multi-well spectrophotometer (Multiskan Go; Thermo Scientific Co., Waltham, MA, USA) with readings acquired at 570 nm and 610 nm. IC_50_ values were calculated by fitting a sigmoidal dose–response curve in GraphPad Prism 8.0.2 (GraphPad Software, San Diego, CA, USA), plotting the logarithmic concentrations of RES and TMZ against viability-associated responses. Data were presented as a percentage relative to the NT group.

#### 4.2.3. Apoptosis Assessment via Flow Cytometry

Early apoptosis, late apoptosis, and necrosis were evaluated in all six U87-MG experimental groups using a NovoCyte Flow Cytometer (ACEA Biosciences, San Diego, CA, USA). Cells assigned to the RES- and TMZ-treated groups were treated with the respective IC_50_ concentrations determined as described in Section 4.2.2, according to the treatment and exposure protocol described in Section 4.2.1. Following the 6 h PEMF or sham exposure, all groups were incubated for an additional 24 h at 37 °C. After incubation, cells were centrifuged at 1500 rpm for 5 min. The cell pellet was stained using the PE Annexin V Apoptosis Detection Kit with 7-AAD (BioLegend, San Diego, CA, USA; Cat. No. 640934) and resuspended in the binding buffer supplied with the kit. Flow-cytometric populations were classified as necrotic (Annexin V−/7-AAD+), late apoptotic (Annexin V+/7-AAD+), viable (Annexin V−/7-AAD−), and early apoptotic (Annexin V+/7-AAD−) cells.

#### 4.2.4. qRT-PCR-Based Profiling of Target Gene Expression

The quantitative real-time PCR (qRT-PCR) was applied to measure the expression levels of Bax, BcL-2, caspase-3, caspase-9, PI3K, AKT, mTOR, and TP53, incorporating slight modifications based on the protocol [67]. Cells were treated with concentrations equivalent to their respective IC_50_ values determined as described in Section 4.2.2, according to the treatment and exposure protocol described in Section 4.2.1. Following the 6 h PEMF or sham exposure, all groups were incubated for an additional 24 h at 37 °C. Total RNA was extracted from the cultured cells using TRIZOL reagent (Invitrogen, Thermo Fisher Scientific, Waltham, MA, USA; Cat. No. 15596026). The quantity and purity of the isolated RNA were assessed with a NanoDrop™ 2000 spectrophotometer (Thermo Scientific, Waltham, MA, USA). Only those RNA samples with an A260/A280 ratio between 1.8 and 2.0 were considered adequate for further analysis. These samples were then used to synthesize complementary DNA (cDNA) using the iScript™ Kit (Bio-Rad, Hercules, CA, USA; Cat. No. 1708891). qRT-PCR was performed using a CFX Connect Real-Time PCR Detection System (Bio-Rad, Hercules, CA, USA) with gene-specific primers listed in Table 1. iTaq™ Universal SYBR Green Supermix (Bio-Rad, Hercules, CA, USA; Cat. No. 1725120) was used for amplification, with GAPDH serving as the reference gene. The thermal cycling program consisted of an initial denaturation at 95 °C for 3 min, followed by 35 cycles of denaturation at 95 °C for 15 s and annealing at 60 °C for 30 s. A melting-curve analysis was performed following amplification to verify the specificity of the amplified products. Relative expression values were obtained using the 2^(−ΔΔCt)^ method.

#### 4.2.5. Protein Expression Analysis by Western Blotting

Cells were seeded in 100 mm Petri dishes at a density of 3 × 10^5^ cells per dish and treated with the respective IC_50_ concentrations of RES and TMZ determined as described in Section 4.2.2, according to the treatment and exposure protocol described in Section 4.2.1. Following the 6 h PEMF or sham exposure, all groups were incubated for an additional 48 h at 37 °C with 5% CO_2_. Cells were collected using a mechanical scraper following two washes with cold PBS. Cells were then centrifuged at 10,000× *g* for 1 min at 4 °C and lysed in 400 μL of RIPA lysis buffer (Santa Cruz Biotechnology, Dallas, TX, USA; Cat. No. sc-364162) supplemented with 1 mM PMSF. Lysates were kept on ice for 5 min and centrifuged at 14,000× *g* for 10 min at 4 °C to obtain a protein-containing supernatant. The resulting supernatants were stored at −80 °C until analysis, and protein concentrations were determined using the Pierce™ BCA Protein Assay Kit (Thermo Scientific, Waltham, MA, USA; Cat. No. 23225). A total of 15 μg of protein per lane was separated on 7.5% to 12% SDS-PAGE gels using a Mini-PROTEAN Tetra Cell apparatus (Bio-Rad, Hercules, CA, USA) and subsequently transferred to PVDF membranes at 100 V for 90 min. Membranes were blocked with TBS-T (0.5 M NaCl, 20 mM Tris-HCl, 0.05% Tween 20) containing 5% non-fat dry milk and exposed to primary antibodies targeting Bax (Abcam, Cambridge, UK; Cat. No. ab32503; 1:2000), BcL-2 (Abcam, Cambridge, UK; Cat. No. ab182858; 1:5000), caspase-3 (Abcam, Cambridge, UK; Cat. No. ab184787; 1:1000), caspase-9 (Abcam, Cambridge, UK; Cat. No. ab32539; 1:1000), and p53 (Abcam, Cambridge, UK; Cat. No. ab179477; 1:2000) for 2 h at room temperature. After washing, membranes were incubated with goat anti-rabbit IgG H&L (HRP)-conjugated secondary antibody (Abcam, Cambridge, UK; Cat. No. ab205718; 1:10,000) for 1 h at room temperature. GAPDH (Abcam, Cambridge, UK; Cat. No. ab22555; 1:5000) was used as the reference protein. Immunoreactive bands were visualized using Clarity™ Western ECL Substrate (Bio-Rad, Hercules, CA, USA; Cat. No. 1705060), and band intensities were quantified using GeneSys imaging software (version 1.9.2, Syngene, Cambridge, UK).

### 4.3. Statistical Processing

Each experiment was independently repeated three times, with three technical replicates included in each independent experiment (*n* = 3 independent experiments). Statistical evaluations were carried out using SPSS software (version 23.0; IBM Corp., Armonk, NY, USA). Data distribution was assessed with the Kolmogorov–Smirnov test. All datasets satisfied the assumption of normality. Homogeneity of variances was assessed using Levene’s test, and all datasets satisfied the assumption of equal variances. Comparisons between two groups were performed using independent *t*-tests, while differences among more than two groups were performed using one-way ANOVA with Tukey’s post hoc analysis. Data are reported as mean values ± standard deviation (SD), and statistical significance was defined at *p* < 0.05.

## 5. Conclusions

Findings demonstrated that RES treatment followed by PEMF exposure was associated with enhanced apoptotic responses in U87-MG glioblastoma cells. The observed upregulation of pro-apoptotic genes and proteins, together with the comparatively limited transcriptional responses of PI3K, AKT, and mTOR, supports a predominantly pro-apoptotic response under the present experimental conditions. However, as the PI3K/AKT/mTOR cascade was evaluated only at the mRNA level, these transcriptional findings cannot establish the functional activation or suppression of this pathway. Further mechanistic and in vivo studies are required to determine the translational potential of this approach.

## Figures and Tables

**Table 1:** The genes’ 5′-3′ forward (F) and 3′-5′ reverse (R) primer sequences.

Gene	Primer Sequences
Bax	F: CCCCCGAGAGGTCTTTTTCC R: TGTCCAGCCCATGATGGTTC
TP53	F: CACATGACGGAGGTTGTGAG R: TAGGGCACCACCACACTATG
Caspase-3	F: AAGGTATGAGGAGGCTGTGC R: ACACAGCCACAGGTATGAGC
Caspase-9	F: AGTTGTCGAAGCCAACCCTA R: TGCTCAGGATGTAAGCCGAC
BcL-2	F: TTCATCGTCCCATCTCCCCT R: ACACACTACAAGTAACACGGCT
PI3K	F: GGAGAACTATGAACAACCTGTG R: CATCTTCCAGTAACGTAGGCAG
AKT	F: CAGGTAGAAGGCAACTCTGCCAAA R: TTCTGCAGCTATGCGCAATGTG
mTOR	F: GCTTGATTTGGTTCCCAGGACAGT R: TGGCCAGCATACCATAGTGAGGTT
GAPDH	F: TGCACCACCAACTGCTTAGC R: AGTTCTATCCGCAGCTCCGCAAT

## Data Availability

The data presented in this study are available within the article and its Supplementary Materials.

## Supplementary Materials

The following supporting information can be downloaded at: https://www.mdpi.com/article/10.3390/ijms27187986/s1. References [68,69,70,71,72,73] are cited in the Supplementary Materials.
