# Supplementary material for: Pulsed Electromagnetic Fields Enhance Resveratrol-Induced Apoptosis Through Modulation of Apoptotic Signaling in U87-MG Glioblastoma Cells
Source: Int J Mol Sci. 2026 Sep 8;27(18):7986. doi: 10.3390/ijms27187986 (PMC13606477; doi:10.3390/ijms27187986)
Supplement: Supplementary file 1 [file ijms-27-07986-s001.zip › ijms-4537742-supplementary.pdf]

Supplementary material

Pulsed Electromagnetic Fields Enhance Resveratrol-Induced Apoptosis Through Modulation of Apoptotic Signaling in U87-MG Glioblastoma Cells

Sinan Kandir [1], Atike Dogan [2], Kayhan Ates [3], Sukru Ozen [3], Serdar Karakurt [4,5] and Cigdem Gokcek-Sarac [2,*]

[1] Department of Veterinary Physiology, Faculty of Ceyhan Veterinary Medicine, Cukurova University, Adana 01250, Türkiye; sinankandir@cu.edu.tr

[2] Department of Biomedical Engineering, Faculty of Engineering, Akdeniz University, Antalya 07058, Türkiye

[3] Department of Electrical and Electronics Engineering, Faculty of Engineering, Akdeniz University, Antalya 07058, Türkiye; kayhanates@akdeniz.edu.tr (K.A.); sukruozen@akdeniz.edu.tr (S.O.)

[4] Department of Biochemistry, Faculty of Science, Selcuk University, Konya 42130, Türkiye; karakurt@selcuk.edu.tr

[5] BioMediTech Institute, Faculty of Medicine and Health Technology, University of Tampere, FI-33014 Tampere, Finland

* Correspondence: cigdemsarac@akdeniz.edu.tr

Details of Experimental Pulsed Magnetic Field Application

The coils were delivered pulsed current by a Pasco 850 Universal Interface. To generate desired signal, Pasco Capstone software (UI-5400) was used, with the pulse frequency set to 75 Hz, a duration of 1.3 milliseconds corresponding to a duty cycle of 1/10, and an amplitude of 15 V. The entire system was enclosed in a custom-built glass cabinet, effectively turning it into an incubator (Figure S1a). Inside, the culture media were kept at a steady 37°C using a precise heating system with temperature sensors for continuous monitoring and adjustments, and no temperature fluctuations above 37°C were observed. A CO_2 infusion system was also integrated to maintain a stable CO_2 concentration of 5%. To minimize moisture loss and create ideal conditions for cell cultures, humidifiers maintained a humidity level of approximately 95%. Before each experiment, the system was sterilized with a 70% ethanol solution and then sealed to prevent unwanted air circulation, while a dedicated power supply ensured continuous and reliable operation of all components, thereby allowing the experiment to mimic the controlled environment of a standard incubator.

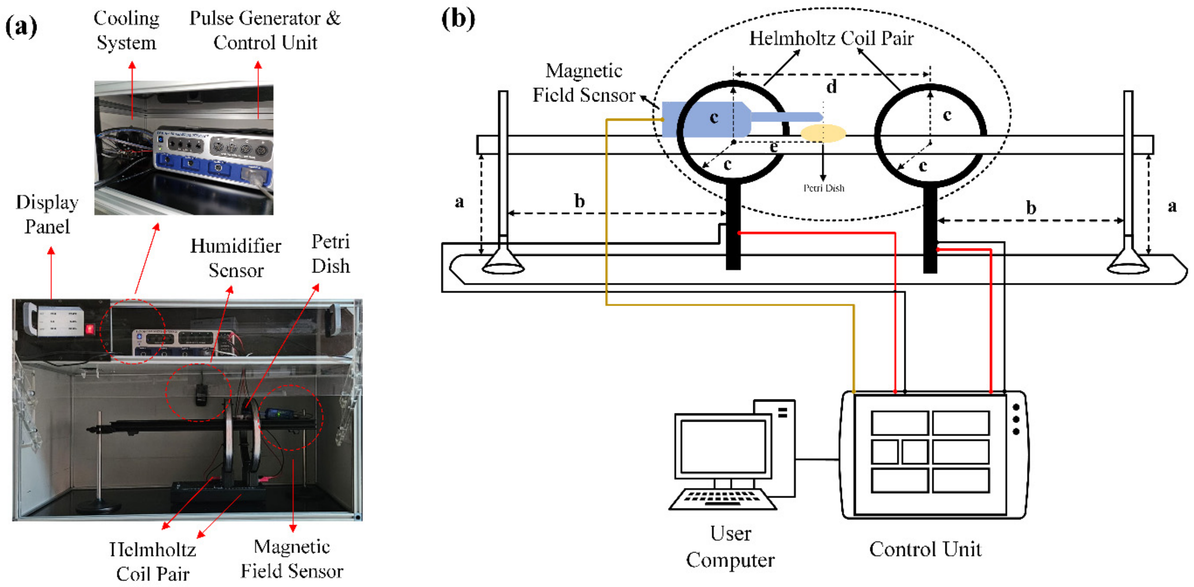

Figure S1. Overview of the exposure system: (a) Experimental exposure setup. (b) Schematic representation with dimensions: a=24 cm, b=30 cm, c=4 cm, d=5 cm, and e=2.5 cm. The Helmholtz coil pair was positioned with a 5 cm gap to maintain uniformity in the magnetic field. A computer-controlled unit was connected to the coils and the magnetic field sensor. Magnetic field measurements were conducted at the midpoint between coils, exactly 2.5 cm from each coil's center. Measurements were repeated 40 times to ensure accuracy, and the

average field strength was recorded using the control software. This figure is adapted from the previous study[15] with minor modifications.

Electromagnetic Dosimetry Simulations

Numerical dosimetry simulations were performed with the finite element method (FEM) based simulation software [65]. The simulation scenario was designed based on prior research [15,66,68]. A three-dimensional model was created to represent a realistic simulation environment, incorporating Helmholtz coil pairs and a petri dish at 75 Hz. An extra-fine mesh level was selected, resulting in 133,122 elements. The outermost boundary of the simulation domain was set as an infinite element domain. The simulation configuration followed the setup illustrated in Fig. S1b. The parameters for the simulation space, including coil and sample characteristics, were derived from previous literature [69-72]. The coil consisted of 500 turns with a radius of 10.06 cm, and the coil wire was modeled as copper with a diameter of 0.64 mm. The sample's magnetic permeability, permittivity, and electrical conductivity were assumed to be 1, 78, and 0.5 S/m, respectively. Figure S2 displays the numerical dosimetry results. The simulations indicated that the peak magnetic flux density between the coils reached 1 mT, aligning with findings from experimental studies. Ates et al. [73] defined the correlation between magnetic flux density, magnetic field, and magnetic permeability. The maximum induced electric field within the petri dish was measured at 1.19 mV/m.

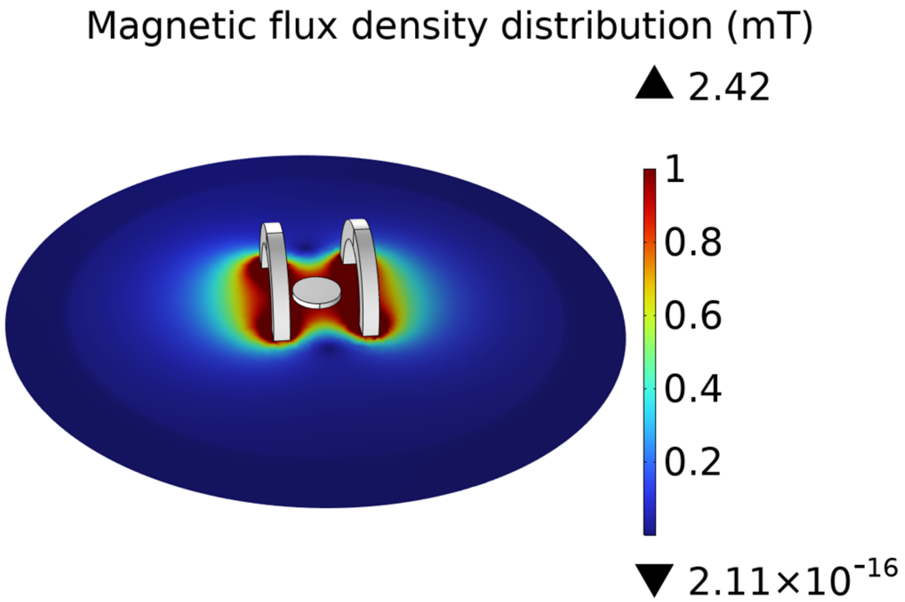

Figure S2. Numerical dosimetry simulation of magnetic flux density distribution generated by Helmholtz coils at 75 Hz.
